# Exploring Self-efficacy for Condom Use in Spanish People: A Trend Analyses by Gender from 2004 to 2020

**DOI:** 10.1007/s10461-022-03937-6

**Published:** 2022-11-28

**Authors:** Cristina Giménez-García, Rafael Ballester-Arnal, Estefanía Ruiz-Palomino, Jesús Castro-Calvo, María Dolores Gil-Llario

**Affiliations:** 1 Departamento de Psicología Básica, Clínica y Psicobiología, Universitat Jaume I, Avda. Vicent Sos Baynat s/n, 12071 Castellón, Spain; 2 Departamento de Personalidad, Evaluación y Tratamientos Psicológicos, Universitat de València, Valencia, Spain; 3 Departamento de Psicología Evolutiva y de la Educación, Universitat de València, Valencia, Spain

**Keywords:** STI, self-efficacy, condom use, gender trend, age

## Abstract

Self-efficacy seems to be one of the most important resources for the different stages underlying condom use and STI prevention. For this reason, this study evaluates trends in self-efficacy by gender, from 2004 to 2008, 2013 and 2020 in Spain. Throughout these years, 6,698 people ranging from 17 to 40 years old, participated filling the Brief scale of condom use self-efficacy. According to our findings, despite the slight improvement in the recent years, self-efficacy still maintains a risky profile for safe sex, especially among the youngest people. Moreover, most of the traditional gender differences continue over the years with women reporting lower scores for condom purchase and men for putting them. However, these differences are not relevant in other dimensions such as using condoms despite drug consumption where women reveal worse results over the years. Therefore, our findings reaffirm the need of intensifying gendered preventive efforts aimed at Spanish people and, particularly, among the youngest.

## Resumen

La autoeficacia parece ser uno de los recursos más importantes para las diferentes etapas que subyacen al uso del preservativo y la prevención de las ITS. Por ello, este estudio evalúa la tendencia de la autoeficacia por género, desde 2004 a 2008, 2013 y 2020 en España. A lo largo de estos años, 6.698 personas con edades comprendidas entre los 17 y los 40 años participaron cumplimentando la Escala Breve de autoeficacia en el uso del preservativo. Según nuestros hallazgos, a pesar de la ligera mejoría en los últimos años, la autoeficacia aún mantiene un perfil de riesgo para el sexo seguro, especialmente entre los más jóvenes. Además, la tradicional diferencia de género continúa a lo largo de los años: las mujeres informan puntuaciones más bajas en la compra de condones y los hombres en ponérselo. Sin embargo, estas diferencias no son relevantes en otras dimensiones como el uso del preservativo a pesar del consumo de drogas donde las mujeres muestran peores resultados a lo largo de los años. Por tanto, nuestros hallazgos reafirman la necesidad de intensificar los esfuerzos preventivos de género dirigidos a los españoles y, en particular, a los más jóvenes.

## Introduction

Sexual transmitted infections (STI) represent a major public health concern, affecting more than 500,000 people in Europe and UK every year [1]. In Spain, with one of the highest rates in Europe, STI prevalence has been growing in the last decades, particularly, affecting young people [2,3]. In this context, despite important progresses on sex education strategies, improving preventive sexual behavior remains a pending issue [3,4]. For this purpose, increasing knowledge about which variables facilitate condom use is necessary.

In this sense, self-efficacy seems to be one of the most important psychosocial factors for sexual risk behavior and condom use barrier [5–10], as well as other barrier methods such as dental dam [11]. That is, those people who report a better self-evaluation about their own ability to condom use are more likely to engage in safer sex behaviors [12–14]. Even more, self-efficacy could mediate the role of other risk factors such as sexual compulsivity or depression [15–17], as well as respond to some barriers such as having sex after drugs consumption that particularly affects condom use [13,18].

Further than a nonspecific construct, in line with proposals such as the Health Action Process [19], self-efficacy involves as many specific self-evaluations as tasks are involved in condom use [20]. For instance, self-efficacy includes a different self-evaluation based on the type of task from planning to knowing how to put on a condom [21,22]. Consequently, people may report differences in self-efficacy for putting condoms (based on mechanic skills), for persuading a partner to use a condom (based on assertiveness in intimacy) or for purchasing condoms (based on the social dimension in unfamiliar contexts). The last one seems to be particularly shameful in younger people [23,24].

Moreover, self-efficacy may reveal differences based on partner context, between casual interactions and regular relationship, in which people practice sexual behaviors with close partners [25]. In this context, concern about gaining the trust of their sexual partners and an emotional connection with them may account for the difference. Therefore, people might report a high self-evaluation of managing condom with regular partners but low self-efficacy to do so with casual partners, and vice versa.

Regarding gender, the literature has revealed some differences that appear to be greater in societies with gender inequality [12,26]. In these contexts, women have reported lower self-efficacy to put on condom and manage sex-partner rejection after condom suggestion [27]. Other studies have revealed the complexity of self-efficacy and gender differences may be dissimilar in each dimension [13,20]. For example, among Spanish young people, men would report more self-confidence for purchasing condoms while women would do it for suggesting condom use.

According to literature, age could also be a relevant variable in the development of HIV prevention self-efficacy [12]. Specifically, in some populations such as Hispanic women [28] or young people from Nigeria [29] levels of condom use self-efficacy would be lower among older people. However, other studies among young people [30,31] have not found statistical differences according to age. Despite these dissimilarities, self-efficacy has demonstrated to be a decisive variable for safe sexual behavior of men and women, in different populations [13–14, 16,32–33].

Probably, for these reasons most of the effective interventions have usually included self-efficacy to prevent sexual transmission of STIs [34–37]. Unfortunately, the literature has not revealed the extent to which self-efficacy might have evolved over the past decade. Consequently, this gap of information complicates the knowledge about riskier profiles, as well as the appropriateness of including self-efficacy in preventive interventions. This would be relevant in conservative cultures such as the Spanish context, where sexuality remains taboo and new infections exceed average European rates [3]. In order to cover this gap of knowledge, this study describes trends in self-efficacy by gender, from 2004 to 2008, 2013 and 2020 among young Spanish people. Based on this aim, five hypotheses were established:

1. Self-efficacy in the Spanish population will not report improvement over the years.
2. Gender differences in self-efficacy will be maintained over the years.
3. Over the years, women will show worse scores than men in self-efficacy related to intimacy and the social domain.
4. Over the years, men will show worse scores than women in self-efficacy related to arousal self-control and mechanical skills.
5. Over the years, young people will show higher scores than older people in self-efficacy.

## Materials and Methods

### Participants

Six thousand six hundred ninety-eight people participated in this study. Regarding gender, 64.3% were women and 35.7% were men. Concerning age, the average was 21.18 (SD = 3.38): 21.52 years old (SD = 3.53) for men and 20.98 years old (SD = 3.28) for women. Relating to sexual experience, 93.8% had already practiced it (93.7% of men and 93.9% of women) and 82.1% were practicing sex at that moment (78.8% of men and 84.1% of women). In addition, 91.1% self-identified as heterosexual (91.7% of men and 90% of women), 6% as bisexual (4.3% of men and 6.9% of women) and 2.9% as homosexual (5.7% of men and 1.3% of women).

### Instrument

The Brief condom use self-efficacy scale is included in The AIDS Prevention Questionnaire [13,38-39] and explores three dimensions of condom use self-efficacy: fear of rejection at the suggestion of using condoms, the ability to manage condom in different scenarios and the acquisition and negotiation of condom. Participants report the level of agreement by seven-item Likert-type measurement from 0 (completely disagree) to 5 (completely agree): (1) I feel or I would feel comfortable when buying condoms; (2) I feel or I would feel comfortable to talk about the use of condoms with a partner before beginning sexual intercourse; (3) If I have to suggest to a partner that we use the condom, I feel or I would feel afraid of him/her rejecting me; (4) I do not feel or I would not feel safe when suggesting the use of condoms to a new partner, because he/she might think that I have an STI; (5) I am sure that I would remember to use the condom although I have consumed alcohol or other drugs; (6) I feel or I would feel uncomfortable when I put the condom on or put it on my partner; (7) I am sure that I could stop even at the moment of greater excitement to put on the condom or put it on my partner. Thus, a score of zero would mean no self-efficacy and a score of five would mean the maximum self-efficacy, having more self-efficacy as they score higher. Additionally, we calculated the sum of these items as the total score ranging from zero as the minimum score (no condom use self-efficacy) to thirty-five as the maximum score (very high self-efficacy for condom use); a higher score means better self-efficacy.

### Procedure

After obtaining the approval from the university’s Research Ethics Committee, from 2004 to 2008, 2013 and 2020, the information was disseminated during activities regarding the World AIDS Day such as giving leaflets, condoms and brief preventive messages by interactive health games placed in common public places for young people.

We motivated young people to join the activities. When they were interested, we gave the information about this research. Once they agreed to participate, they gave us their informed consent and filled out the Brief condom use self-efficacy scale [13,38-39] anonymously and voluntarily. In this process, trained psychologists clarified doubts and guaranteed its rigor. Once the questionnaire was completed, they participated in interactive health games.

The evaluation procedure was identical each year to deal with potential confounding variables, except in 2020. Because of COVID-19, we disseminated the study and developed the activities by the online social networks targeting young people. There are no data from 2009 to 2012, as well as from 2014 to 2019, because of logistical problems in conducting the field research.

**Fig. 1:**
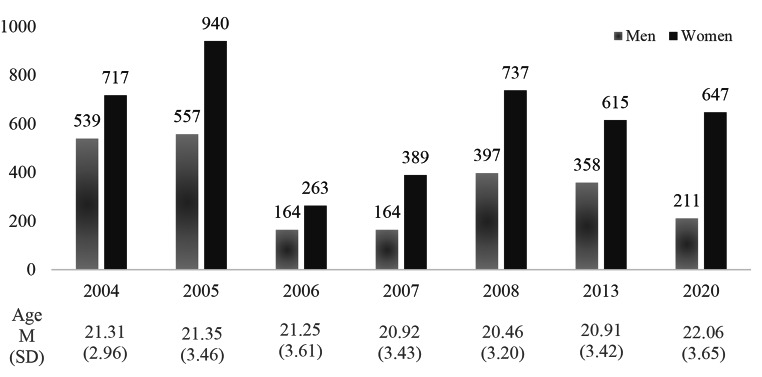
Number of participants by gender and age per year of recruitment

Initially, 6,723 people were interested, but only 99.6% of them (n = 6,698) participated because of the eligibility criteria: being native-Spanish speaker and from 17 to 40 years. The recruitment, between 2004 and 2008, 2013 and 2020 showed a similar distribution of gender participation by year (see Fig. 1). This was in line with the gender distribution of Spanish people where women exceed men [40].

### Analyses

Initially, we performed the Analyses of variance and the Bonferroni correction to evaluate whether self-efficacy revealed differences from 2004 to 2020 for all participants and by gender. In addition, we performed Student’s t and Cohen’s d to examine whether there were differences between men and women in each year. Moreover, we carried out the linear regression to analyze if gender and age were relevant for self-efficacy in each year, as well as for its global trend from 2004 to 2020. For the last one, we included the analyses of interaction between sex*year and age*year.

## Results

**Fig. 2:**
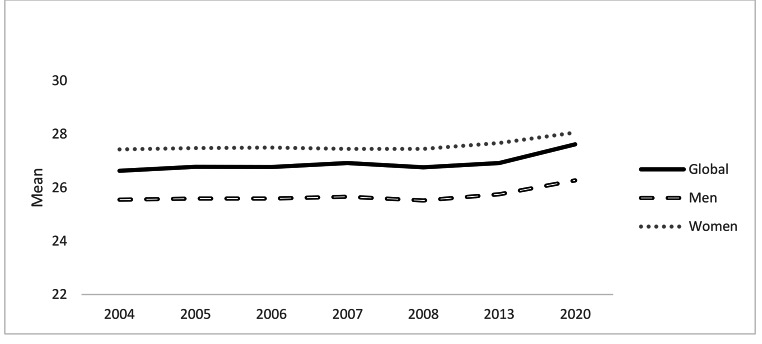
Unstandardized predicted value of Global Self-efficacy and by gender

**Fig. 3:**
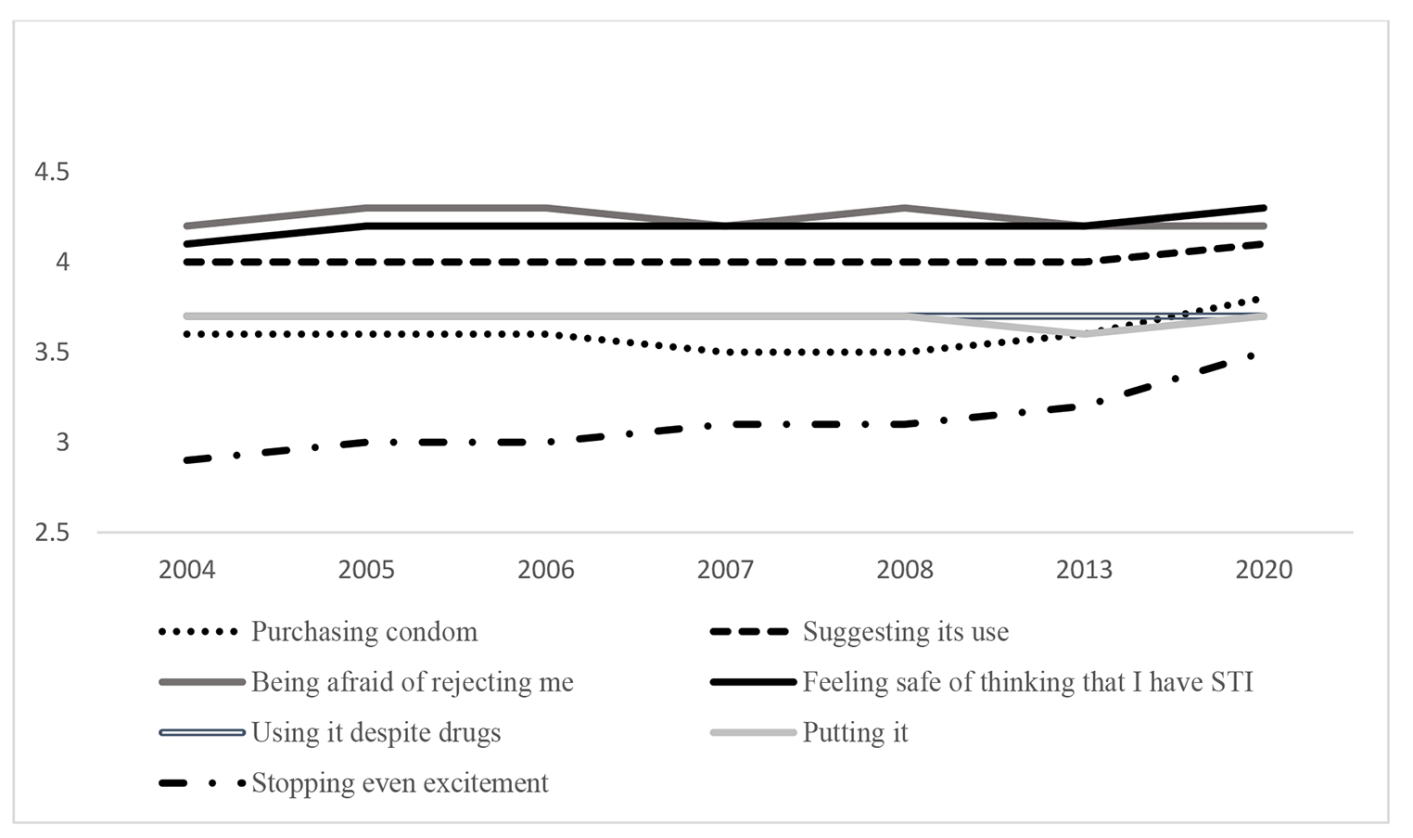
. Unstandardized predicted value of Self-efficacy dimensions by year

Firstly, regarding general scores, all the analyses reveal statistically significant differences by ANOVA except for using condom despite drug consumption. The total score of self-efficacy seems to decrease in 2008 and 2013 although exceeds the earlier results in 2020, revealing differences by Bonferroni (see Table 1; Fig. 2). In particular, scores range between 26.33 (in 2008) and 28.15 (in 2020). Concerning specific dimensions, self-evaluation about being afraid of him/her rejecting me shows the highest value while stopping even at the moment of greater excitement and purchasing condoms show the lowest results (see Fig. 3). The last one, suggesting condom use, purchasing condom and feeling safe about he/she might think that I have an STI show the highest scores in 2020 revealing differences by Bonferroni. In case of suggesting condom use and feeling safe of thinking that I have an STI also reveal high scores in the earlier evaluations (2005 and 2006). Moreover, being afraid of him/her rejecting me and putting condom the highest scores are in 2004 and 2006–2007 respectively.

**Table 1:** Differential analyses of self-efficacy per year

Variable	2004 M(SD)	2005 M(SD)	2006 M(SD)	2007 M(SD)	2008 M(SD)	2013 M(SD)	2020 M(SD)	Total	F(p)	Bonferroni
Self-efficacy (0–35)	26.72 (5.19)	27.09 (5.10)	26.97 (5.37)	26.93 (5.31)	26.33 (5.27)	26.36 (5.29)	28.15 (4.96)	26.90 (5.22)	11.86 (0.000)	2005 > 2008, 2013 2020 > 2004–2008,2013
Purchasing condoms (0–5)	3.54 (1.53)	3.63 (1.50)	3.51 (1.58)	3.69 (1.52)	3.64 (1.46)	3.71 (1.43)	3.76 (1.48)	3.64 (1.50)	2.61 (0.016)	2020 > 2004
Suggesting condom use (0–5)	4.02 (1.30)	4.10 (1.22)	3.99 (1.29)	4.09 (1.22)	3.95 (1.28)	3.89 (1.32)	4.29 (1.21)	4.04 (1.27)	8.78 (0.000)	2005 > 2013 2020 > 2004–2006,2008,2013
Being afraid of rejecting me (0–5)	4.39 (1.15)	4.33 (1.20)	4.33 (1.20)	4.27 (1.33)	4.09 (1.37)	4.23 (1.32)	4.30 (1.19)	4.28 (1.25)	6.51 (0.000)	2008 < 2004–2006, 2020
Feeling safe of thinking that I have an STI	4.21 (1.41)	4.32 (1.30)	4.40 (1.24)	4.25 (1.38)	4.07 (1.49)	4.09 (1.46)	4.50 (1.06)	4.24 (1.36)	11.35 (0.000)	2005 > 2008, 2013 2006 > 2008, 2013 2020 > 2004, 2007, 2008, 2013
Using condom despite drugs (0–5)	3.71 (1.46)	3.78 (1.38)	3.66 (1.53)	3.76 (1.39)	3.76 (1.38)	3.63 (1.44)	3.77 (1.31)	3.73 (1.41)	1.55 (0.155)	
Putting condom (0–5)	3.79 (1.62)	3.76 (1.60)	3.85 (1.59)	3.85 (1.61)	3.60 (1.69)	3.59 (1.76)	3.84 (1.76)	3.74 (1.65)	3.80 (0.000)	
Stopping even excitement	3.01 (1.81)	3.11 (1.71)	3.00 (1.73)	2.96 (1.73)	3.14 (1.71)	3.10 (1.67)	3.67 (1.46)	3.14 (1.71)	15.44 (0.000)	2020 > 2004–2008, 2013

**Fig. 4:**
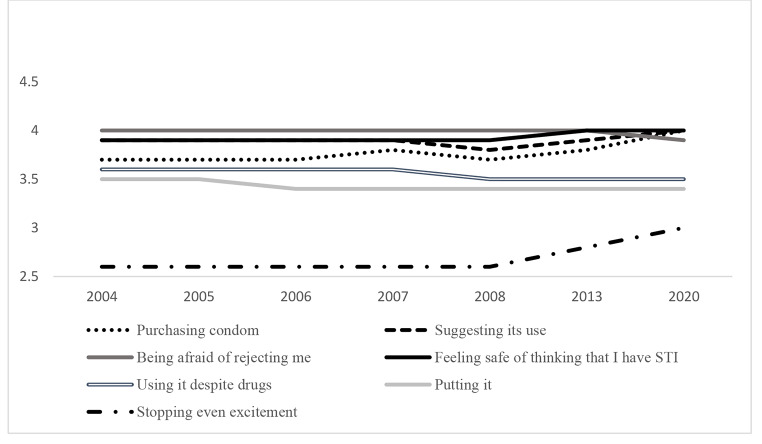
Unstandardized predicted value of Self-efficacy dimensions by year for men

**Fig. 5:**
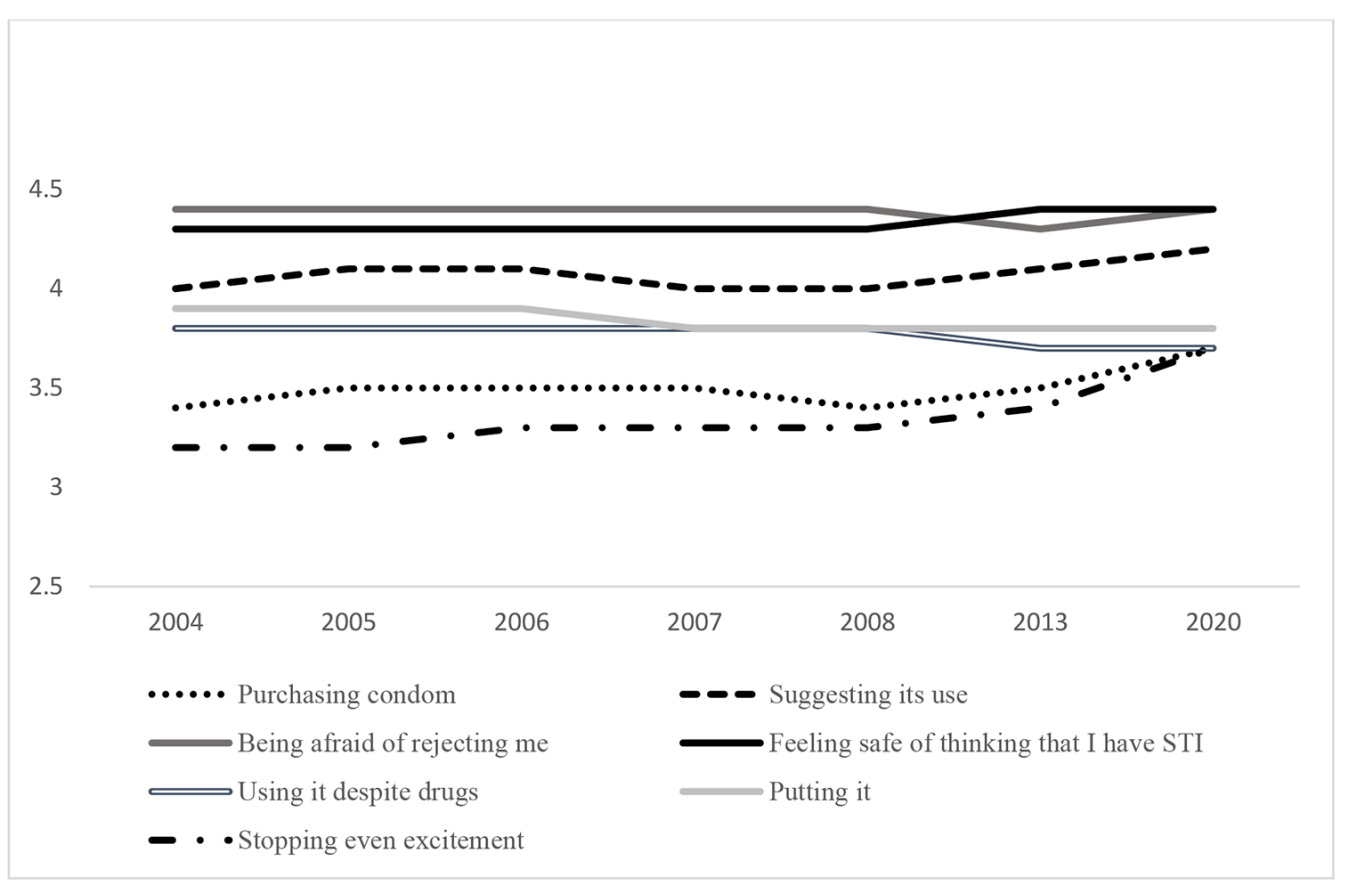
Unstandardized predicted value of Self-efficacy dimensions by year for women

Differentiating trends by gender (see Table 2; Figs. 4 and 5), for both men and women the total score of self-efficacy shows statistically significant differences by Bonferroni correction. The highest scores are in 2020 although women also show higher results in 2005.

**Table 2:** Differences in self-efficacy by gender per year

Variable	Sex	2004	2005	2006	2007	2008	2013	2020	TOT	F(p)	Bonf
Self-efficacy (0–35)	Men	25.44 (5.25)	25.78 (5.49)	25.70 (5.42)	25.66 (5.69)	25.23 (5.57)	25.32 (5.22)	27.28 (5.27)	25.66 (5.42)	3.65 (0.001)	2020 > 2004–2005, 2008, 2013
	Women	27.65 (4.94)	27.86 (4.69)	27.75 (5.21)	27.46 (5.06)	26.94 (5.00)	26.95 (5.24)	28.42 (4.84)	27.57 (4.98)	6.93 (0.000)	2005;2020 > 2008, 2013
	t (p)	7.36 (0.000)	7.30 (0.000)	3.67 (0.000)	3.42 (0.001)	5.10 (0.000)	4.55 (0.000)	2.75 (0.006)	13.76 (0.000)		
	d(CI)	0.44 0.32;0.55	0.42 0.31;0.52	0.39 0.18;0.59	0.34 0.15;0.53	0.33 0.20; 0.45	0.31 0.18;0.45	0.23 0.01;0.39	0.37 0.32;0.42		
Purchasing condoms (0–5)	Men	3.76 (1.43)	3.80 (1.47)	3.56 (1.61)	4.04 (1.38)	3.90 (1.34)	3.86 (1.34)	3.76 (1.51)	3.82 (1.43)	2.03 (0.058)	
	Women	3.38 (1.58)	3.53 (1.50)	3.49 (1.56)	3.53 (1.55)	3.49 (1.51)	3.63 (1.48)	3.76(1.48)	3.54 (1.52)	3.87 (0.001)	2020 > 2004, 2008
	t (p)	-4.49 (0.000)	-3.35 (0.001)	-0.46 (0.646)	-3.79 (0.000)	-4.80 (0.000)	-2.50 (0.013)	-0.02 (0.984)	-7.31 (0.000)		
	d(CI)	-0.25 − 0.37;-0.13	-0.18 − 0.29;-0.01		− 0.34 − 0.53;-0.15	-0.28 − 0.41;-0.16	0.16 0.03; 0.29		− 0.18 − 0.23;-0.13		
Suggesting condom use (0–5)	Men	3.87 (1.33)	3.99 (1.24)	3.94 (1.28)	4.03 (1.24)	3.86 (1.31)	3.74 (1.38)	4.23 (1.32)	3.92 (1.31)	3.54 (0.002)	2020 > 2004, 2008, 2013
	Women	4.12 (1.27)	4.17 (1.21)	4.01 (1.31)	4.12 (1.21)	4.00 (1.26)	3.98 (1.28)	4.31 (1.17)	4.11 (1.24)	5.00 (0.000)	2020 > 2006, 2008, 2013
	t (p)	3.31 (0.001)	2.76 (0.006)	0.53 (0.592)	0.82 (0.409)	1.73 (0.084)	2.72 (0.007)	7.97 (0.426)	5.65 (0.000)		
	d (CI)	0.19 0.08;0.31	0.22 0.11;0.32				0.18 0.05;0.32		0.15 0.09;0.20		
Being afraid of rejecting me (0–5)	Men	4.18 (1.26)	4.08 (1.35)	4.03 (1.43)	3.85 (1.60)	3.92 (1.47)	4.05 (1.41)	4.21 (1.25)	4.06 (1.38)	2.29 (0.032)	
	Women	4.54 (1.03)	4.48 (1.06)	4.51 (1.01)	4.46 (1.15)	4.18 (1.31)	4.34 (1.26)	4.33 (1.16)	4.40 (1.16)	8.00 (0.000)	2008 < 2004–2007 2013,2020 < 2004
	t (p)	5.36(0.000)	5.92 (0.000)	3.64 (0.000)	4.37 (0.000)	2.83 (0.005)	3.12 (0.002)	1.28 (0.200)	9.87 (0.000)		
	d (CI)	0.32 0.20;0.43	0.34 0.23;0.45	0.37 0.16;0.58	0.47 0.28;0.66	0.19 0.06;0.32	0.22 0.09;0.35		0.27 0.22;0.32		
Feeling safe of thinking that I have an STI (0–5)	Men	3.94 (1.55)	4.15 (1.32)	4.26 (1.34)	3.88 (1.55)	3.79 (1.61)	3.82 (1.56)	4.24 (1.23)	3.99 (1.48)	5.03 (0.000)	2008 < 2005, 2006,2020 2013 < 2005, 2020
	Women	4.41 (1.26)	4.42 (1.27)	4.49 (1.17)	4.41 (1.27)	4.22 (1.40)	4.25 (1.38)	4.58 (0.99)	4.39 (1.27)	5.81 (0.000)	2005 > 2008 2020 > 2008, 2013
	t (p)	5.67 (0.000)	3.82 (0.000)	1.79 (0.074)	3.86 (0.000)	4.40 (0.000)	4.28 (0.000)	3.42 (0.001)	10.86 (0.000)		
	d (CI)	0.34 0.22;0.45	0.21 0.10;0.32		0.39 0.20;0.58	0.29 0.16;0.42	0.30 0.16;0.43	0.32 0.16;0.49	0.29 0.24;0.34		
Using condom despite drug consumption (0–5)	Men	3.53 (1.54)	3.59 (1.44)	3.47 (1.58)	3.76 (1.35)	3.64 (1.48)	3.60 (1.43)	3.66 (1.40)	3.60 (1.47)	0.85 (0.528)	
	Women	3.85 (1.39)	3.88 (1.34)	3.78 (1.48)	3.76 (1.41)	3.83 (1.32)	3.64 (1.45)	3.81 (1.27)	3.80 (1.37)	2.19 (0.040)	2013 < 2005
	t (p)	3.75 (0.000)	3.83 (0.000)	1.96 (0.051)	-0.04 (0.968)	1.92 (0.055)	0.38 (0.704)	1.35 (0.175)	5.50 (0.000)		
	d (CI)	0.21 0.10;0.33	0.21 0.10;0.31						0.14 0.09;0.19		
Putting condom (0–5)	Men	3.58 (1.66)	3.61 (1.61)	3.46 (1.76)	3.47 (1.74)	3.24 (1.79)	3.28 (1.81)	3.72 (1.72)	3.48 (1.72)	3.45 (0.002)	2008 < 2005, 2020
	Women	3.94 (1.56)	3.85 (1.59)	4.09 (1.42)	4.01 (1.52)	3.80 (1.61)	3.77 (1.71)	3.87 (1.65)	3.88 **(**1.60)	2.11 (0.049)	
	t (p)	3.84 (0.000)	2.86 (0.004)	3.74 (0.000)	3.43 (0.001)	5.14 (0.000)	4.09(0.000)	1.05 (0.290)	9.12 (0.000)		
	d (CI)	0.22 0.11;0.34	0.15 0.04;0.26	0.38 0.18;0.59	0.34 0.15;0.53	0.32 0.19;0.35	0.28 0.14;0.41		0.19 0.14;0.24		
Stopping even excitement (0–5)	Men	2.47 (1.82)	2.57 (1.76)	2.70 (1.78)	2.53 (1.72)	2.79 (1.80)	2.85 (1.74)	3.44 (1.51)	2.70 (1.77)	8.51 (0.000)	2020 > 2004–2008, 2013 2013 > 2004
	Women	3.41 (1.69)	3.43 (1.60)	3.17 (1.67)	3.14 (1.71)	3.34 (1.62)	3.24 (1.62)	3.74 (1.44)	3.38 (1.62)	7.88 (0.000)	2020 > 2004–2008, 2013
	t (p)	9.20 (0.000)	9.29 (0.000)	2.67 (0.008)	3.79 (0.000)	4.93 (0.000)	3.39 (0.001)	2.43 (0.015)	15.17 (0.000)		
	d (CI)	0.59 0.47;0.70	0.52 0.41;0.63	0.27 0.48;0.06	0.36 0.17;0.54	0.33 0.20;0.45	0.23 0.10;0.37	0.21 0.04;0.37	0.40 0.35;0.45		

For men all dimensions of self-efficacy show statistically significant differences except for purchasing condoms and using condom despite drug consumption. In line with Bonferroni, suggesting condom use and stopping even at the moment of greater excitement show the highest results in 2020. Similarly, in putting condom and feeling safe about he/she might think that I have an STI men report higher scores in 2020 but also in 2005 or 2006.

About women, they have shown statistically significant differences for all dimensions of self-efficacy. In line with the Bonferroni correction, purchasing and suggesting condom use, stopping even at the moment of greater excitement and feeling safe about he/she might think that I have an STI women show the highest score in 2020 being the lowest in 2004, 2013, 2007 and 2008 respectively. However, in using condom despite drug consumption and being afraid of him/her rejecting me, women reveal the best scores from 2004 to 2006.

In relation to gender differences, women exceed men in scores of total self-efficacy, although the low effect size by the Cohen’s d diminishes over the years. Similarly, gender reveals statistically significant differences over the years in stopping even at the moment of greater excitement, but the Cohen’s d results diminish from medium to low effect size. This dimension of self-efficacy and feeling safe about he/she might think that I have an STI are the only ones in which women still exceed men scores in 2020 revealing statistically significant differences. In other dimensions such as suggesting condom use and using it despite drug consumption, women also exceed men but in earlier years. Contrarily, men report higher scores in purchasing condoms.

For both men and women, the two items about being afraid of him/her rejecting me show the highest result while stopping even at the moment of greater excitement reveals the lowest. About the unstandardized predicted value, Figs. 4 and 5 show how self-efficacy about purchasing condoms or suggesting its use would show a slight increase, while using condom despite drugs consumption would decrease for both men and women.

Regarding the linear regression in each year (see Table 3), gender and age seem to play a role over the years in the total score of self-efficacy except for 2020 in which age is excluded: women and older people would report higher scores. Concerning gender, women would report higher scores in stopping even at the moment of greater excitement and feeling safe about he/she might think that I have an STI, as well as in the earlier evaluations of using condom despite drug consumption and suggesting its use. In addition, women would report more self-efficacy in being afraid of him/her rejecting me and putting condom except in 2020. In contrast, men would report higher scores for purchasing condom except for 2006 and 2020. In general, the explained variance is low ranging from 1 to 8%. About age, older people are more likely to show higher scores in purchasing and suggesting condoms, as well as in putting condoms in 2005, 2006 and 2013 and in stopping even excitement from 2005 to 2007. However, age seem not to be relevant for the other dimensions of self-efficacy.

**Table 3:** Linear regression of self-efficacy by age and sex in each year

		2004		2005		2006		2007		2008		2013		2020	
		B; e	CI	B; e	CI	B; e	CI	B; e	CI	B; e	CI	B; e	CI	B; e	CI
Self-efficacy (0–35)	Age	0.15; 0.05	0.05;0.25	0.14;0.03	0.06;0.21	0.35;0.07	0.20;0.49	0.22;0.07	0.08;0.36	0.11;0.05	0.01;0.21	0.18;0.04	0.08;0.28	0.08;0.04	− 0.01;0.18
	Sex	-2.29;0.30	-2.88;-1.71	-2.16;0.27	-2.69;-1.62	-2.16;0.54	-3.23;-1.10	-2.04;0.50	-3.03;-1.05	-1.75;0.33	-2.41;-1.09	-1.69;0.35	-2.38;-0.99	-1.22;0.41	-2.40;-0.40
		r^2^ = 0.051 F(p) = 31.75 (0.000)		r^2^ = 0.048 F(p) = 36.02(0.000)		r^2^ = 0.088 F(p) = 18.28(0.000)		r^2^ = 0.04 F(p) = 11.44(0.000)		r^2^ = 0.028 F(p) = 15.53(0.000)		r^2^ = 0.036 F(p) = 14.43(0.000)		r^2^ = 0.013 F(p) = 5.28(0.005)	
Purchasing condoms (0–5)	Age	0.06;0.01	0.03;0.09	0.04;0.01	0.02;0.06	0.08;0.02	0.04;0.12	0.06;0.01	0.02;0.10	0.03;0.01	0.003;0.05	0.05;0.01	0.02;0.08	0.04;0.01	0.01;0.07
	Sex	0.34;0.08	0.17;0.51	0.02;0.08	0.08;0.40	0.04;0.15	− 0.26;0.35	0.43;0.14	0.16;0.71	0.39;0.09	0.20;0.58	0.21;0.09	0.02;0.40	− 0.04;0.12	− 0.28;0.20
		r^2^ = 0.032 F(p) = 20.68(0.000)		r^2^ = 0.019 F(p) = 14.25(0.000)		r^2^ = 0.036 F(p) = 7.74(0.000)		r^2^ = 0.043 F(p) = 12.24(0.000)		r^2^ = 0.023 F(p) = 11.36(0.000)		r^2^ = 0.023 F(p) = 11.32(0.000)		r^2^ = 0.01 F(p) = 3.91(0.020)	
Suggesting condom use (0–5)	Age	0.05;0.01	0.03;0.07	0.02;0.01	0.01;0.04	0.06;0.01	. 03;0.09	0.04;0.01	0.01;0.07	0.05;0.01	0.03;0.08	0.05;0.01	0.02;0.07	− 0.01;0.01	− 0.04;0.00
	Sex	− 0.28;0.07	− 0.42;-0.13	− 0.19;0.06	− 0.32;-0.06	− 0.09;0.13	− 0.34;0.16	− 0.14;0.11	− 0.37;0.07	− 0.16;0.08	− 0.32;0.004	− 0.26;0.08	− 0.43;-0.09	− 0.06;0.10	− 0.26;0.13
		r^2^ = 0.024 F(p) = 14.94(0.000)		r^2^ = 0.011 F(p) = 7.96(0.000)		r^2^ = 0.033 F(p) = 6.93(0.001)		r^2^ = 0.018 F(p) = 5.08(0.007)		r^2^ = 0.023 F(p) = 11.33(0.000)		r^2^ = 0.025 F(p) = 12.07(0.000)		r^2^ = 0.004 F(p) = 1.51(0.220)	
Being afraid of rejecting me (0–5)	Age	0.00;0.06 − 0.02;0.02		0.01;0.01 − 0.01; 0.02		0.03;0.01 0.00;0.06		0.02;0.01 − 0.01;0.05		− 0.01;0.01 − 0.03;0.01		− 0.01;0.01 − 0.02;0.02		0.01;0.01 − 0.01; 0.03	
	Sex	− 0.36; 0.06 − 0.49;-0.23		− 0.40;0.06 − 0.53;-0.28		− 0.49;0.12 − 0.73;-0.25		− 0.63;0.12 − 0.87;-0.38		− 0.25;0.08 − 0.42;-0.08		-2.86;0.08 − 0.46;-0.11		− 0.14;0.10 − 0.33;0.05	
		r^2^ = 0.02 F(p) = 15.26 (0.000)		r^2^ = 0.02 F(p) = 20.50 (0.000)		r^2^ = 0.05 F(p) = 10.85 (0.000)		r^2^ = 0.046 F(p) = 13.07(0.000)		r^2^ = 0.009 F(p) = 4.69(0.009)		r^2^=. 0.01 F(p) = 5.17(0.006)		r^2^=. 0.004 F(p) = 1.54(0.213)	
Feeling safe of thinking that I have an STI (0–5)	Age	0.01;0.01 − 0.02;0.02		− 0.01;0.01 − 0.03;0.01		− 0.01;0.01 − 0.03;0.03		0.01;0.01 − 0.02; 0.04		− 0.03;0.01 − 0.03;0.02		0.01;0.01 − 0.01;0.04		− 0.05;0.01 − 0.02;0.01	
	Sex	− 0.47;0.08 − 0.63;-0.31		− 0.26;0.07 − 0.40;-0.12		− 0.23;0.12 − 0.48;0.01		− 0.54;0.12 − 0.80;-0.29		− 0.43;0.09 − 0.61;-0.24		− 0.43;0.09 − 0.63;-0.24		− 0.33;0.01 − 0.50;-0.15	
		r^2^ = 0.02 F(p) = 17.07(0.000)		r^2^ = 0.01 F(p) = 8.37(0.000)		r^2^=0.01 F(p) = 1.72 (0.180)		r^2^ = 0.03 F(p) = 9.04(0.000)		r^2^ = 0.02 F(p) = 10.56(0.000)		r^2^ = 0.02 F(p) = 10.55(0.000)		r^2^ = 0.02 F(p) = 7.33(0.001)	
Using condom despite drugs (0–5)	Age	0.01;0.01	− 0.02;0.03	0.01;0.01	− 0.01;0.03	− 0.004;0.02	− 0.04;0.03	0.01;0.01	− 0.01;0.05	− 0.01;0.01	− 0.04;0.01	0.01;0.01	− 0.01;0.03	0.01;0.01	− 0.01;0.03
	Sex	− 0.32;0.08	− 0.49;-0.16	− 0.29;0.07	− 0.44;-0.15	− 0.30;0.15	− 0.61;0.00	− 0.01;0.13	− 0.27;0.24	− 0.18;0.09	− 0.36;-0.001	− 0.04;0.09	− 0.23;0.15	-0.1;0.1	− 0.37;0.06
		r^2^ = 0.012 F(p) = 7.42(0.001)		r^2^ = 0.01 F(p) = 8.04(0.000)		r^2^ = 0.10 F(p) = 1.93(0.146)		r^2^ = 0.002 F(p) = 0.45(0.634)		r^2^ = 0.006 F(p) = 3.06 (0.047)		r^2^ = 0.000 F(p) = 0.220(0.803)		r^2^ = 0.003 F(p) = 1.06 (0.346)	
Putting condoms (0–5)	Age	0.01;0.01	− 0.01;0.04	0.02;0.01	0.00;0.04	0.07;0.02	0.02;0.11	0.02;0.02	− 0.01;0.06	0.01;0.01	− 0.01;0.05	0.04;0.01	0.01;0.07	0.03;0.01	− 0.00;0.06
	Sex	− 0.36;0.09	− 0.55;-0.18	− 0.26;0.08	− 0.43;-0.09	− 0.65;0.15	− 0.96;-0.33	− 0.57;0.15	− 0.87;-0.27	− 0.56;0.11	− 0.78;-0.34	− 0.50;0.11	− 0.73;-0.23	− 0.17;0.14	− 0.45;0.09
		r^2^ = 0.01 F(p) = 8.02(0.000)		r^2^ = 0.008 F(p) = 5.99(0.003)		r^2^ = 0.062 F(p) = 13.21 (0.000)		r^2^ = 0.027 F(p) = 7.46(0.001)		r^2^ = 0.026 F(p) = 13.13(0.000)		r^2^ = 0.024 F(p) = 11.96(0.000)		r^2^ = 0.003 F(p) = 2.20(0.111)	
Stopping even excitement (0–5)	Age	0.02;0.01 − 0.04;0.06		0.03;0.01 0.01;0.06		0.05;0.02 0.03;0.09		0.05;0.02 0.01;0.09		0.04;0.01 − 0.02;0.03		0.01;0.01 − 0.02;0.03		0.01;0.01 − 0.01;0.04	
	Sex	− 0.95;0.10 -1.15;-0.75		− 0.87;0.09 -1.05;-0.70		− 0.48;0.17 − 0.83;-0.14		− 0.67;0.16 − 0.99;-0.35		− 0.54;0.10 − 0.75;-0.33		− 0.39;0.11 − 0.61;-0.17		− 0.31;0.12 − 0.55;-0.07	
		r^2^ = 0.07 F(p) = 44.81(0.000)		r^2^ = 0.06 F(p) = 49.84(0.000)		r^2^ = 0.03 F(p) = 5.78(0.003)		r^2^ = 0.03 F(p) = 10.34 (0.000)		r^2^=0.02 F(p) = 12.92(0.000)		r^2^ = 0.01 F(p) = 6.10(0.0002)		r^2^ = 0.01 F(p) = 3.53 (0.030)	

Additionally, the linear regression analyses about general scores (see Table 4) reveal how women and older people are more likely to report higher scores in the total score of self-efficacy, suggesting condoms, being afraid of him/her rejecting me, putting condoms and stopping even at the moment of greater excitement. However, men and older people are more likely to purchase condoms. In case of feeling safe about he/she might think that I have an STI and using condom despite drug consumption the age seems not to play an important role.

**Table 4:** Linear regression: significant coefficients of self-efficacy by sex, age, year and interactions

Variables		B	e	CI	F (p)	r [2]
Self-efficacy	Age	0.17	0.01	0.13;0.21	97.25 (0.000)	0.044
	Sex	-1.97	0.13	-2.24;-1.71		
	Year	0.03	0.01	0.007; 0.056		
Purchasing condoms	Age	0.05	0.00	0.04;0.06	51.92 (0.000)	0.023
	Sex	0.26	0.03	0.18;0.33		
	Year	0.01	0.00	0.00;0.01		
Suggesting condom use	Age	0.04	0.00	0.03; 0.04	37.32 (0.000)	0.017
	Sex	− 0.20	0.03	− 0.27;-0.14		
Being afraid of rejecting me	Age	0.010	0.005	0.001; 0.019	39.57 (0.000)	0.018
	Sex	− 0.34	0.03	− 0.41; − 0.28		
	Year	− 0.008	0.003	− 0.01;-0.002		
Feeling safe of thinking that I have an STI	Sex	− 0.39	0.035	− 0.46; − 0.32	43.81 (0.000)	0.020
Using condom despite drug	Sex	− 0.21	0.03	− 0.28;-0.13	10.99 (0.000)	0.005
Putting condoms	Age	0.00	0.01	0.02;0.04	38.93 (0.000)	0.018
	Sex	− 0.42	0.04	− 0.50;-0.33		
Stopping even excitement	Age	0.027	0.006	0.015; 0.039	104.16 (0.000)	0.046
	Sex	− 0.668	0.044	− 0.75; − 0.58		
	Year	0.027	0.004	0.019; 0.035		

Regarding the time of evaluation, people from the latest evaluations are more likely to report higher scores in total self-efficacy, self-efficacy about purchasing condoms and stopping even at the moment of greater excitement. However, people from the earliest evaluations are more likely to report higher scores in self-efficacy about being afraid of him/her rejecting me. For the other dimensions, the time of evaluation seems not to play an important role. In addition, the interactions between sex*year and age*year were excluded from the model.

## Discussion

Firstly, our findings support a risk profile of self-efficacy for STI prevention. The total self-efficacy has slightly improved over the years but revealed worse results than other studies focused on Spanish population [13]. This is important considering the relevance of self-efficacy for condom use in different contexts and populations [14,41].

About specific dimensions, self-efficacy about being afraid of somebody rejecting me would obtain worse results over the years. Probably, difficulties to eliminate stigma and prejudices about STIs may facilitate this tendency, in particular, for HIV-AIDS [43]. This may be related to be afraid of STI shame and rejection and, consequently, would affect the self-evaluation of condom use [43–44].

In addition, self-efficacy to suggest or put on condom in a sexual relationship and to use condom despite drug consumption have reported lower results than in other Spanish population [13] and have not improved over the years. First, the weight of relationship values still prevalent in Hispanic cultures, based on romanticism, passion, and assumed fidelity, would decrease self-efficacy for condom management in intimacy [45–46]. Second, lower scores related drug consumption have already been well supported in relation to condomless sex, interfering some cognitive mechanisms that may affect self-efficacy [47–48]. This result is especially concerning due to the high prevalence of alcohol and other drugs use among Spanish people [49].

On the other hand, purchasing condoms and stopping even at the moment of greater excitement seem to improve over the years although reveal the worst results. As some studies pointed out, the first improvement could be partially related to the higher anonymity of current commercial settings that would reduce embarrassment, which is associated with less condom purchase [23,50−51]. Thus, situational factors out of personal control may modulate this perception but no other personal variables such as social skills and fear of negative evaluation. In terms of individual factors, their perceived ability to manage safer sex during higher arousal also reveals difficulties. According to past studies, a higher sexual arousal moderates cognitive ability and decreases the perceived probability of using condoms, and consequently may reduce the self-efficacy perceived [41,52].

Regarding gender differences in total self-efficacy, as we hypothesized, men and women would maintain the dissimilarities showed in past studies even these would slightly diminish [27]. In particular, women report higher self-efficacy scores except for purchasing condoms in which men exceed women. This difference, in line with past studies [50], may be related to traditional gender characters that hinders women’s public spaces, making difficult their self-protection against STI exposure [53–54]. In the case of men, self-efficacy to put on a condom would reveal the lowest results compared to women. As past studies described, men would report higher sexual sensation seeking and arousal and, consequently, more difficulties to use condom under higher sexual excitement what may influence on self-efficacy [55–57].

On the other hand, other dimensions such as suggesting condom use and using condom despite drug consumption have not maintained these gender differences over years. In line with some authors [58], women would be less likely to negotiate and use condoms under alcohol use that seems to increase in the recent decade for Spanish young women [59]. Additionally, women would get worse results over the years in being afraid of somebody rejecting me. Probably, gendered socialization of sexuality would increase social desirability and shame among women, showing more difficulties to manage some social contexts and the related self-efficacy [53–54].

In relation to the hypothesis about age, younger people would report more difficulties to develop safer sex behaviors, as past studies have stated [60–61], except for using condom despite drug consumption and feeling safe of thinking that I have an STI at the suggestion of using condoms. These last results would support previous findings [29] in which aging did not necessarily increase some dimensions of self-efficacy maybe because of their particular difficulty. Therefore, policies should target younger people to strengthen their self-efficacy and facilitate health behaviors [62]. However, these policies should consider specific dimensions of self-efficacy that seem to require particular attention at different developmental stages.

At this point, some limitations should be considered to analyze these findings. First, the self-reported measure might increase social desirability even though it has revealed adequate psychometric characteristics. Second, the gap of evaluation makes more difficult to generalize these results. Additionally, regardless of the large number of participants, the percentage of different sexual orientations per year and gender is scarce to carry out an analysis about their specific evolution. In addition, the relationship that self-efficacy could establish with other variables such as sexual assertiveness should be analyzed to assess whether they are modulating its influence. Both aspects should be studied in the future.

## Conclusion

Beyond these aspects, these findings add relevant information about the statement of self-efficacy among Spanish people. In particular, the risky tendency seems to be maintained especially for younger people, except for some dimensions such as condom use after drug consumption that is extremely relevant due to the rates of alcohol consumption in Spain. In addition, gender differences are maintained, with women reporting higher scores except for condom purchase, as well as the most important decrease in condom use despite drug use. Therefore, our findings reaffirm the need to intensify gender preventive efforts aimed at younger Spanish people and, particularly, in some dimensions of self-efficacy such as sexual self-control, fear of negative evaluation and drugs consumption.

## Data Availability

Not applicable.
